# Heme oxygenase 1 alleviates nonalcoholic steatohepatitis by suppressing hepatic ferroptosis

**DOI:** 10.1186/s12944-023-01855-7

**Published:** 2023-07-08

**Authors:** Xiwei Yuan, Lu Li, Ying Zhang, Rong Ai, Dongdong Li, Yao Dou, Mengmeng Hou, Dandan Zhao, Suxian Zhao, Yuemin Nan

**Affiliations:** Department of Traditional and Western Medical Hepatology, Hebei Provincial Key Laboratory of liver fibrosis in chronic liver diseases, Third Hospital of Hebei Medical University, Shijiazhuang, 050051 Hebei China

**Keywords:** Nonalcoholic steatohepatitis, Heme oxygenase-1, Ferroptosis, Lipid peroxidation, iron metabolism

## Abstract

**Background:**

Heme oxygenase 1 (HO-1) has an influential but insufficiently investigated effect on ferroptosis, which is a novel form of programmed cell death and may play an effect on nonalcoholic steatohepatitis (NASH). However, the understanding of the mechanism is limited. Herein, our study aimed to explore the mechanism and role of HO-1 in NASH ferroptosis.

**Methods:**

Hepatocyte conditional HO-1 knockout (HO-1^HEPKO^) C57BL/6J mice were established and fed a high-fat diet (HFD). Additionally, wild-type mice were fed either a normal diet or a HFD. Hepatic steatosis, inflammation, fibrosis, lipid peroxidation, and iron overload were assessed. AML12 and HepG2 cells were used to investigate the underlying mechanisms in vitro. Finally, liver sections from NASH patients were used to clinically validate the histopathology of ferroptosis.

**Results:**

In mice, HFD caused lipid accumulation, inflammation, fibrosis, and lipid peroxidation, which were aggravated by HO-1^HEPKO^. In line with the *in vivo results*, HO-1 knockdown upregulated reactive oxygen species accumulation, lipid peroxidation, and iron overload in AML12 and HepG2 cells. Additionally, HO-1 knockdown reduced the GSH and SOD levels, which was in contrast to HO-1 overexpression in vitro. Furthermore, the present study revealed that the NF-κB signaling pathway was associated with ferroptosis in NASH models. Likewise, these findings were consistent with the liver histopathology results of NASH patients.

**Conclusion:**

The current study showed that HO-1 could alleviate NASH progression by mediating ferroptosis.

**Supplementary Information:**

The online version contains supplementary material available at 10.1186/s12944-023-01855-7.

## Introduction

Nonalcoholic fatty liver disease (NAFLD), which affects approximately 25% of adults worldwide, is the most predominant cause of chronic liver disease at present and has emerged as a significant public health issue [1]. In 2020, the international consensus modified the diagnostic criteria for NAFLD and redefined it as metabolic dysfunction-associated fatty liver disease (MAFLD) [2]. Because the utility of MAFLD has been insufficiently tested in the real world, we still use the concept of NAFLD in this study. Nonalcoholic steatohepatitis (NASH) is a severe form of NAFLD characterized by hepatic steatosis, lobular inflammation, and ballooning degeneration, with or without any fibrosis [3]. Moreover, it may progress to cirrhosis and even liver cancer [3]. It is commonly believed that NAFLD is associated with numerous factors, such as lipid metabolism disorders, inflammation, oxidative stress, insulin resistance (IR), and endoplasmic reticulum stress [4]. Despite a number of studies being conducted, the exact mechanisms underlying NASH pathogenesis remain incompletely understood. Notably, emerging findings have revealed that ferroptosis may potentially be involved in the occurrence and progression of liver diseases, including NAFLD [5–8].

Ferroptosis is a new type of nonapoptotic regulated cell death characterized by abnormal lipid peroxidation and iron metabolism [9]. Experimentally, iron homeostasis disorder will produce reactive oxygen species (ROS) via the Fenton reaction. Consequently, those ROS could be changed into hydroxyl radicals and undergo peroxidation reactions with lipids to trigger ferroptosis [10]. Additionally, the depletion of glutathione (GSH) and inactivity of glutathione peroxidase 4 (GPX4) are prominent biochemical changes in ferroptosis, and the system Xc-/GSH/GPX4 axis plays an important role in this process [10]. Notably, lipid peroxidation caused by ferroptosis can be reversed by some small molecule compounds (such as vitamin E and ferrostatin-1 (Fer-1)), while others (such as erastin and sulfasalazine) can induce ferroptosis [10, 11]. Moreover, increased ROS and lipid peroxidation are significant elements in ferroptosis occurrence and are commonly believed to play critical roles in the pathological processes of NASH [4]. Thus, using antioxidant substances to control ferroptosis and subsequently intervene in the progression of NASH is highly significant and necessary.

The primary and rate-limiting enzyme of heme metabolism, heme oxygenase-1 (HO-1), catalyzes the breakdown of heme into biliverdin, free iron, and carbon monoxide and has profound antioxidant and anti-inflammatory properties [12]. In this regard, HO-1 is commonly known to have phylactic power in many human diseases, including NAFLD and iron metabolism disorders [13–15]. Studies have shown that patients with HO-1 gene mutations and mice with HO-1 knockout cannot reuse iron and exhibit iron accumulation, particularly in the hepatic parenchyma, which contributes to oxidative damage, tissue injury, and persistent inflammation [12]. However, the effect of HO-1 on ferroptosis is highly controversial, and the role of ferroptosis in the pathophysiology of NASH needs further investigation.

In the current study, we discussed the influence of ferroptosis on NASH and discovered a meaningful role of HO-1 in ferroptosis using hepatocyte conditional HO-1 knockout (HO-1^HEPKO^) mice, HO-1 knockdown and overexpression cells, and NASH patients, revealing HO-1 as a hopeful target for the prevention and treatment of hepatic steatohepatitis by inhibiting ferroptosis.

## Materials and methods

### Animals and treatments

All animal experiments were conducted following the guidelines of the Hebei Committee for Care and Use of Laboratory Animals and were given the go-ahead by the Animal Experimentation Ethics Committee of the Third Hospital of Hebei Medical University, China. All mice were kept in a specific pathogen-free controlled environment with a 12-hour cycle of light and dark and unrestricted access to a conventional water and chow diet.

Utilizing CRISPR‒Cas9 technologies, HO-1^HEPKO^ mice with a C57BL/6J background were produced [16]. To induce experimental steatohepatitis and fibrosis, eight-week-old male C57BL/6J wild type (WT) and matched HO-1^HEPKO^ mice (n = 6/group) were randomly fed either a 42% fat high fat diet (HFD, TD. 88,137, Harlan Teklad, North America) for 32 weeks. Additionally, a normal diet (ND) was fed to control WT mice. At the end of the experiment, all mice were sacrificed under isoflurane anesthesia, and blood and organs were collected for subsequent analysis.

### Western blot analysis

Using RIPA buffer supplemented with PMSF, 20 mg of liver tissue was used for protein extraction. PVDF membranes were used to transfer the proteins separated by SDS‒PAGE. After blocked for 1 h in TBST with 5% skim milk, membranes were incubated with antibodies against reduced glyceraldehyde-phosphate dehydrogenase (GAPDH, 1:2000, AB0036, Abways Technology, Shanghai, China), β-Actin (1:1000, AB0035, Abways Technology, Shanghai, China), HO-1 (1:2000, ab189491, Abcam, Cambridge, US), heterodimeric protein containing a light chain (SLC7A11, 1:1000, CY7046, Abways Technology, Shanghai, China), GPX4 (1:3000, CY6959, Abways Technology, Shanghai, China), Ferritin (1:500, CY5396, Proteintech Group, Illinois, USA), acyl-CoA synthetase long-chain family member 4 (ACSL4, 1:1000, CY10198, Abways Technology, Shanghai, China), lipoxygenase (LOX, 1:500, CY6864, Abways Technology, Shanghai, China), nuclear factor kappa-B (NF-κB, 1:2000, CY5040, Abways Technology, Shanghai, China), NF-kappa-B inhibitor alpha (IKBα, 1:1000, CY5026, Abways Technology, Shanghai, China), inhibitor of kappa B kinase beta (IKKβ, 1:1000, ab124957, Abcam, Cambridge, US), interleukin-6 (IL-6, 1:1000, ab259341, Abcam, Cambridge, US), interleukin-1 beta (IL-1β, 1:800, 26,048-a-AP, Proteintech Group, Illinois, USA), tumor necrosis factor-alpha (TNFα, 1:1000, ab215188, Abcam, Cambridge, US), Lysyl oxidase homolog 2 (LOXL2, 1:1000, CY7106, Abways Technology, Shanghai, China), alpha-smooth muscle actin (α-SMA, 1:2000, CY1132, Abways Technology, Shanghai, China) and transforming growth factor beta (TGFβ,1:1000, CY2179, Abways Technology, Shanghai, China) overnight at 4 °C. Following incubation, membranes were probed with secondary antibodies Dylight800 goat anti-rabbit or anti-mouse IgG (1:7000, A23910, Abbkine Scientific, Wuhan, China) at 37 °C for 1 h. Finally, the Odyssey fluorescence imaging system (LI-COR, USA) was used to detect the fluorescence protein bands, and densitometry was analyzed with ImageJ software (NIH, Bethesda, MD, USA).

### Histological evaluation

Liver tissues fixed in 4% neutral formaldehyde were embedded in paraffin blocks. Hematoxylin-eosin (H&E) and Masson’s trichrome staining were used to stain 4 μm-thick liver sections. The sum of steatosis, lobular inflammation, and fibrosis was assessed by two expert liver pathologists for NASH assessment using the NAFLD activity score (NAS) algorithm. To analyze lipid accumulation, Oil Red O staining was performed using frozen tissue sections counterstained with hematoxylin.

### Immunohistochemistry staining

For immunohistochemistry staining, primary antibodies against HO-1 (1:2000, ab189491, Abcam, Cambridge, US), GPX4 (1:100, ab125066, Abcam, Cambridge, US), ACSL4 (1:200, ab155282, Abcam, Cambridge, US), NF-κB (1:100, CY5040, Abways Technology, Shanghai, China), IKKβ (1:100, ab124957, Abcam, Cambridge, US) and a DAB staining solution (polymer method) kit (PV6000D, ZSGB-BIO, Beijing, China) containing a horseradish peroxidase (HRP)-conjugated secondary antibody and DAB working solution were used. Furthermore, image analysis and quantification were performed using the Image-Pro Plus v6.0 program (Media Cybernetics, MD, USA).

### Biochemical analysis

For biochemical analysis, mouse feed and water were removed the night before the experiment and at least 8 h of fasting. All mice were euthanized, blood was collected, and serum alanine aminotransferase (ALT), aspartate aminotransferase (AST), total cholesterol (TC), triglyceride (TG), and fasting blood glucose were measured using appropriate enzymatic kits in a dedicated autoanalyzer (Olympus AU270, Tokyo, Japan).

### Cell culture

Mouse immortalized hepatocytes (AML12) and human hepatocytes (HepG2) were purchased from Procell Life Science & Technology Co., Ltd. AML12 cells were cultured in Dulbecco’s modified Eagle’s medium (DMEM)/Ham’s F12 supplemented with 10% fetal bovine serum (FBS), a mixture of insulin-transferrin-selenium, 0.1 mM dexamethasone, 100 U/mL penicillin and 100 µg/mL streptomycin. HepG2 cells were grown in DMEM with 10% FBS, 100 units of penicillin and 100 µg streptomycin per ml. The cells were maintained at 5% CO_2_ and 37 °C in a moist environment.

### Cell treatment

To establish in vitro models of NASH, AML12 and HepG2 cells were exposed to free fatty acids (FFAs), including oleic acid and palmitic acid (OA: PA = 2:1), for 24 h at a concentration of 300 µM. Simultaneously, cells were separately treated with erastin (8 µM) and Fer-1 (4 µM), which were obtained from Med Chem Express (Shanghai, China). We also established an HO-1 knockdown model by transfecting small interfering RNA (siRNA) into cells while inducing HO-1 overexpression with pcDNA3.1 HO-1 plasmid (Gene Pharma, Shanghai, China).

Cells were divided into 6 groups to investigate the role of HO-1 in hepatocytes. Namely, the control group (NC group, nontreatment), model group (FFA group, 300 µM FFA treatment), ferroptosis inducer group (Erastin group, 8 µM erastin intervention), FFA combined with ferroptosis inhibitor group (Fer-1 group, 4 µM Fer-1 intervention, 300 µM FFA treatment), HO-1 knockdown group (kdHO-1 group, HO-1 siRNA transfection, 300 µM FFA treatment), HO-1 overexpression group (oeHO-1 group, pcDNA3.1 HO-1 plasmid transfection, 300 µM FFA treatment).

#### Oil Red O staining of hepatocytes

AML12 and HepG2 cells were dyed with Oil Red O (G1260, Solarbio, Beijing, China) in accordance with the manufacturer’s instructions to view the lipid droplets. Briefly, they were incubated in 12-well plates, intervened as mentioned in the preceding paragraph, particularly pertaining to cell treatment, including FFA intervention, erastin and Fer-1 treatments. then fixed for 30 min with 4% paraformaldehyde solution. After thoroughly rinsing in distilled water, the cells were colored for 10 min with Oil Red O, counterstained for 3 min with hematoxylin, sealed with glycerin gelatin, observed under the microscope, and analyzed by Image-Pro Plus v6.0 software (Media Cybernetics, MD, USA).

### Measurement of MDA, 4-HNE, GSH, and SOD in Liver Tissues and cells

The contents of hepatic and intracellular lipid peroxidation were determined using malondialdehyde (MDA) assay kits (A003-1-2, Jiancheng, Nanjing, China) and 4-hydroxynonenal (4-HNE) ELISA kits (E-EL-0128c, Elabscience, Wuhan, China) in accordance with the manufacturer’s instructions. Additionally, hepatic and intracellular GSH and superoxide dismutase (SOD) were determined using commercial GSH and SOD quantification assay kits (A006-2-1, A001-3-2, Jiancheng, Nanjing, China) following the manufacturer’s directions.

### Lipid ROS assessment

The lipid peroxidation probe C11 BODIPY 581/591 (D3861, Invitrogen, Shanghai, China) was used to evaluate cellular lipid ROS. Briefly, cells were seeded 12 h before treatment in a 6-well plate with DMEM. Following treatment, the cells were rinsed three times with phosphate-buffered saline (PBS) before adding fluorescent dyes (final concentration: 2.5 M) to the complete medium and incubating for 30 min at 37 °C. Furthermore, Hoechst 33,342 was used to stain the nuclei. After washing three times with PBS, the intracellular fluorescence was measured (IX53, Olympus, Japan).

### Iron assay

Colorimetric assay kits were used to determine the total and ferrous iron contents in liver tissue and hepatocytes according to the manufacturer’s recommendations (E-BC-K772-M, E-BC-K773-M, Elabscience, Wuhan, China). A Ferrorange fluorescent probe (F374, Dojindo, Shanghai, China) was used to locate ferrous ions in living cells, and Hoechst 33,342 was utilized to stain the nuclei. Finally, the fluorescence was observed by fluorescence microscopy (IX53, Olympus, Japan).

### Human samples

Twenty-one patients with NASH and 6 healthy subjects were recruited from the Third Hospital of Hebei Medical University. All of the NASH patients underwent liver biopsy, and the control liver samples were collected from liver transplant donors. The demographic characteristics of the subjects are listed in Table 1. The Ethics Committee of Third Hospital of Hebei Medical University approved the study (ke 2021-085-1). All subjects signed informed consent forms.

### Statistical analysis

Statistical analysis was carried out using SPSS 26.0 (IBM SPSS Statistics, Armonk, USA) and GraphPad Prism Software (GraphPad Software, La Jolla, CA, USA). One-way analysis of variance (ANOVA), Student’s *t* test and the Mann‒Whitney *U* test were used to evaluate differences between groups. Data are expressed as the mean ± standard deviation (SD) or medians and interquartile range (IQR). A statistically significant *P* value of less than 0.05 was evaluated.

## Results

### HO-1 deficiency aggravates HFD-induced NASH and lipid droplet accumulation

**Fig. 1:**
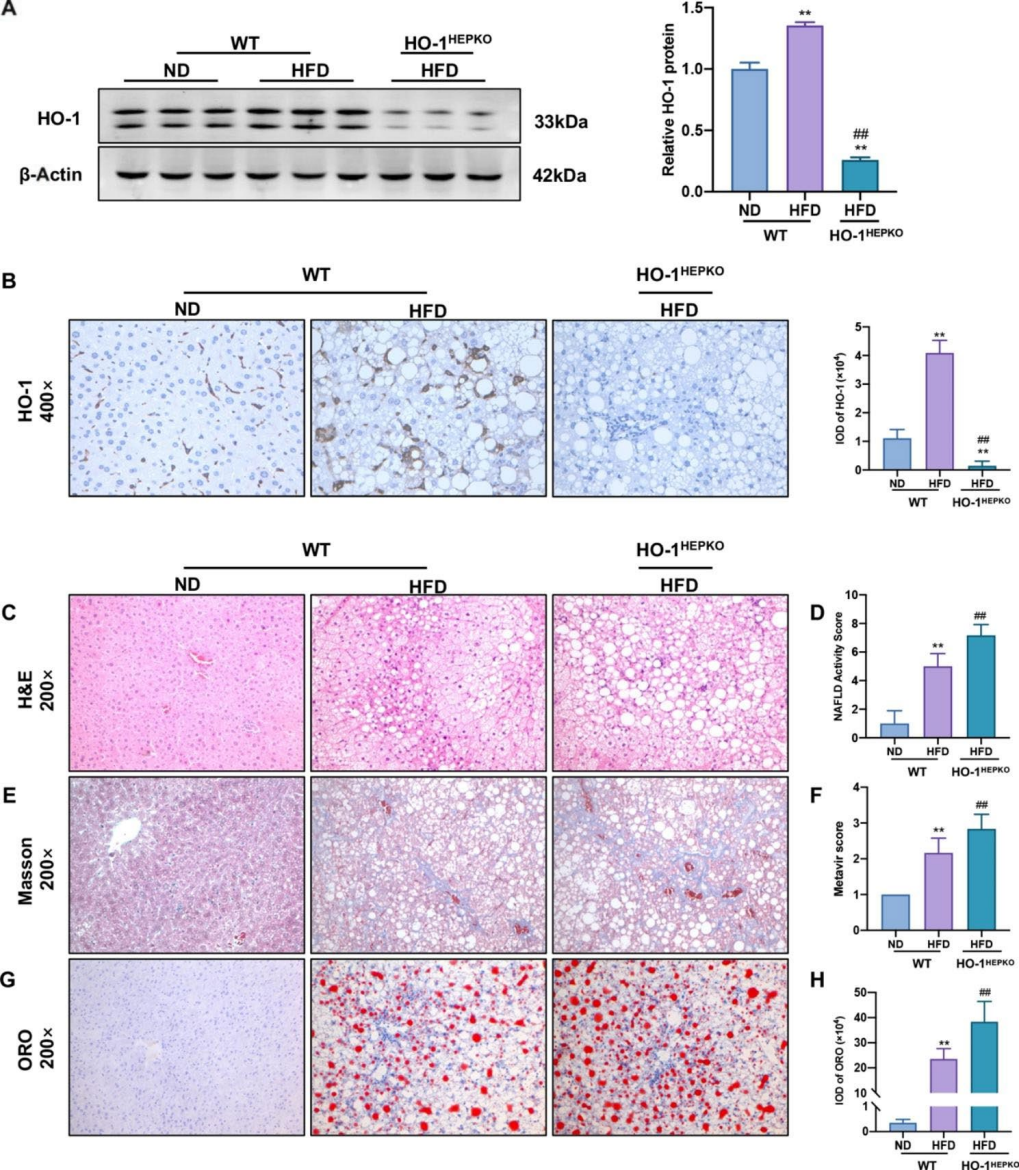
Hepatocyte conditional knockout of HO-1 aggravates HFD-induced NASH. **(A)** Western blot analysis of HO-1 in liver tissue. β-Actin served as the loading control. **(B)** Images of HO-1 (×400 magnification) staining of representative liver sections in WT and HO-1^HEPKO^ mice. **(C)** Images of H&E (×200 magnification) staining of representative liver sections and **(D)** the NAFLD activity score. **(E)** Images of Masson (×200 magnification) staining of representative liver sections and **(F)** the Metavir score. **(G, H)** Images and IOD of Oil Red O (×200 magnification) staining of representative liver sections were obtained from each group. Values are the mean ± SD (n = 6 per group). *P < 0.05, **P < 0.01 compared to the WT ND group; *#P <* 0.05, *##P* < 0.01 compared to the WT HFD group

To estimate the underlying effect of HO-1 on NASH occurrence, we fed WT and HO-1^HEPKO^ mice a HFD for 32 weeks to establish steatohepatitis, while WT control mice were fed a ND. As shown in Fig. 1A, HO-1 levels in the liver tissue were obviously increased in the WT HFD group compared with the WT ND group and virtually absent in the HO-1^HEPKO^ HFD group. Likewise, immunohistochemical staining showed that hepatic HO-1 expression was consistent with the above findings (Fig. 1B). Histopathological changes were assessed in the three groups utilizing H&E, Masson, and Oil Red O staining. Importantly, the WT HFD group showed pronounced hepatic steatosis, ballooning, inflammation, and fibrosis compared to the WT ND group. By comparison, the HO-1^HEPKO^ HFD group showed apparently more aggravated changes than the WT HFD group (Fig. 1C F). As demonstrated by Oil Red O staining (Fig. 1G H), the WT HFD group produced significantly more lipid droplets than the WT ND group. Furthermore, the accumulation of lipid droplets in the HO-1^HEPKO^ HFD group was considerably higher than that in the WT HFD group.

### Effect of HO-1 deficiency on biochemical parameters and liver injury in mice

**Fig. 2:**
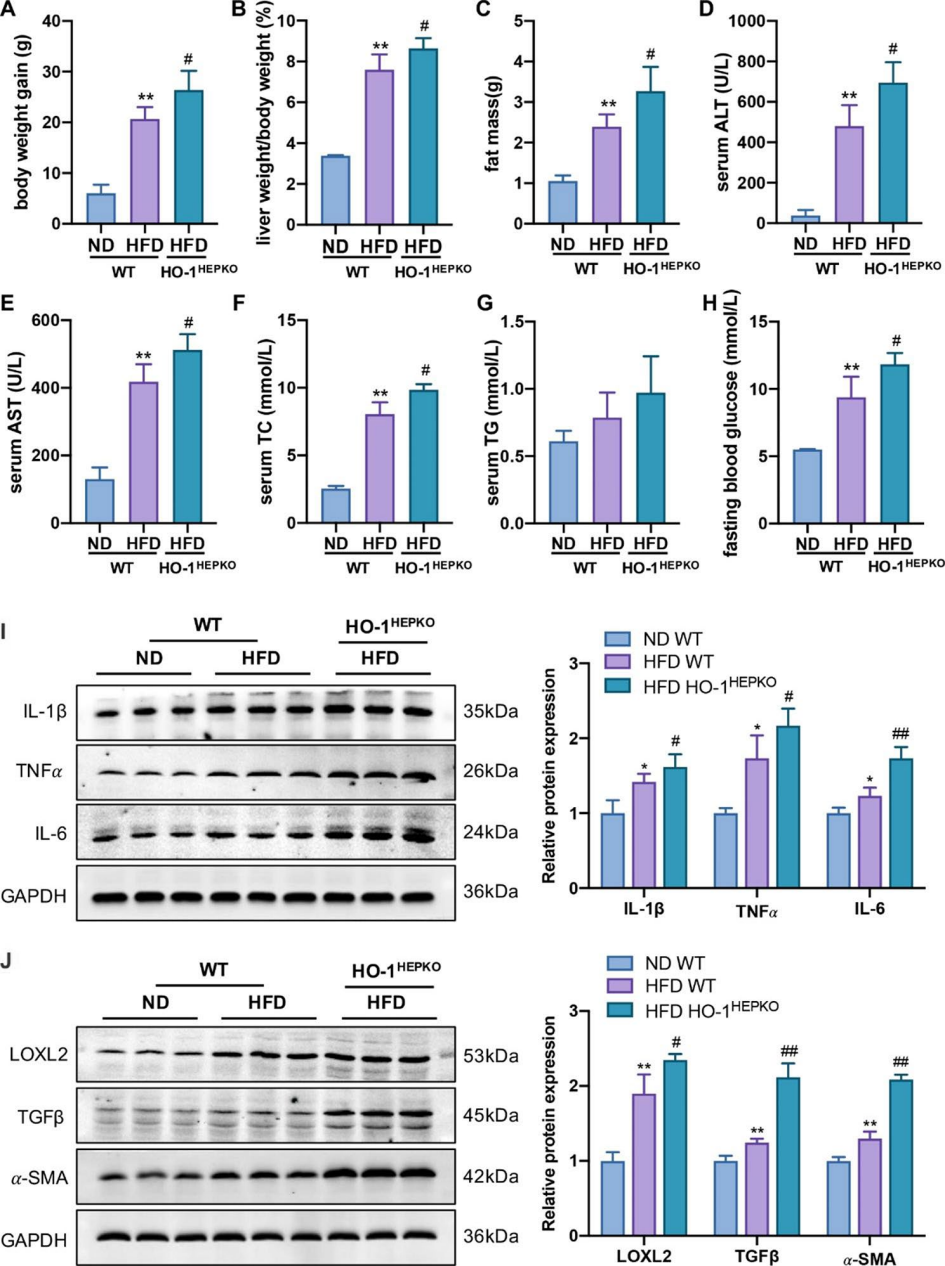
Deficiency of HO-1 exacerbates HFD-fed mice in terms of biochemical parameters and liver injury. **(A)** Body weight gain, **(B)** liver weight/body weight, and **(C)** fat mass in WT and HO-1^HEPKO^ mice fed normal chow and HFD. Serum **(D)** ALT, **(E)** AST, **(F)** TC, **(G)** TG, and **(H)** fasting blood glucose in each group. **(I)** Western blot analysis of IL-6, IL-1β, and TNFα in liver tissue. **(J)** Western blot analysis of LOXL2, α-SMA, and TGFβ in liver tissue. Values are the mean ± SD (n = 6 per group). *P < 0.05, **P < 0.01 compared to the WT ND group; *#P <* 0.05, *##P* < 0.01 compared to the WT HFD group

In accordance with the above findings, the WT HFD group exhibited increased body weight gain, liver-to-body weight ratio, and fat mass compared to the WT ND group, which were augmented after HO-1 deficiency (Fig. 2A C). Additionally, the levels of serum ALT, AST, total TC, and fasting blood glucose in the WT HFD group were higher than those in the WT ND group. Likewise, HO-1 knockout aggravated the increased ALT, AST, TC, and fasting blood glucose levels (Fig. 2D F, 2 H). The levels of serum TG in the three groups showed an increasing trend, but there were no differences (Fig. 2G). To gain additional insight into the impact of HO-1 on NASH progression, we detected the protein levels of key factors related to inflammation and fibrosis. HO-1 deficiency significantly increased the protein levels of inflammation-associated genes (e.g., IL-6, IL-1β, and TNFα) and fibrosis-associated genes (e.g., LOXL2, α-SMA, and TGFβ) in the HO-1^HEPKO^ HFD group compared with the WT HFD group (Fig. 2I J). In short, these findings revealed that hepatocyte HO-1 deficiency significantly exacerbated the progression of NASH in mice fed a HFD.

### HO-1 knockout exacerbates ferroptosis in mice with NASH

**Fig. 3:**
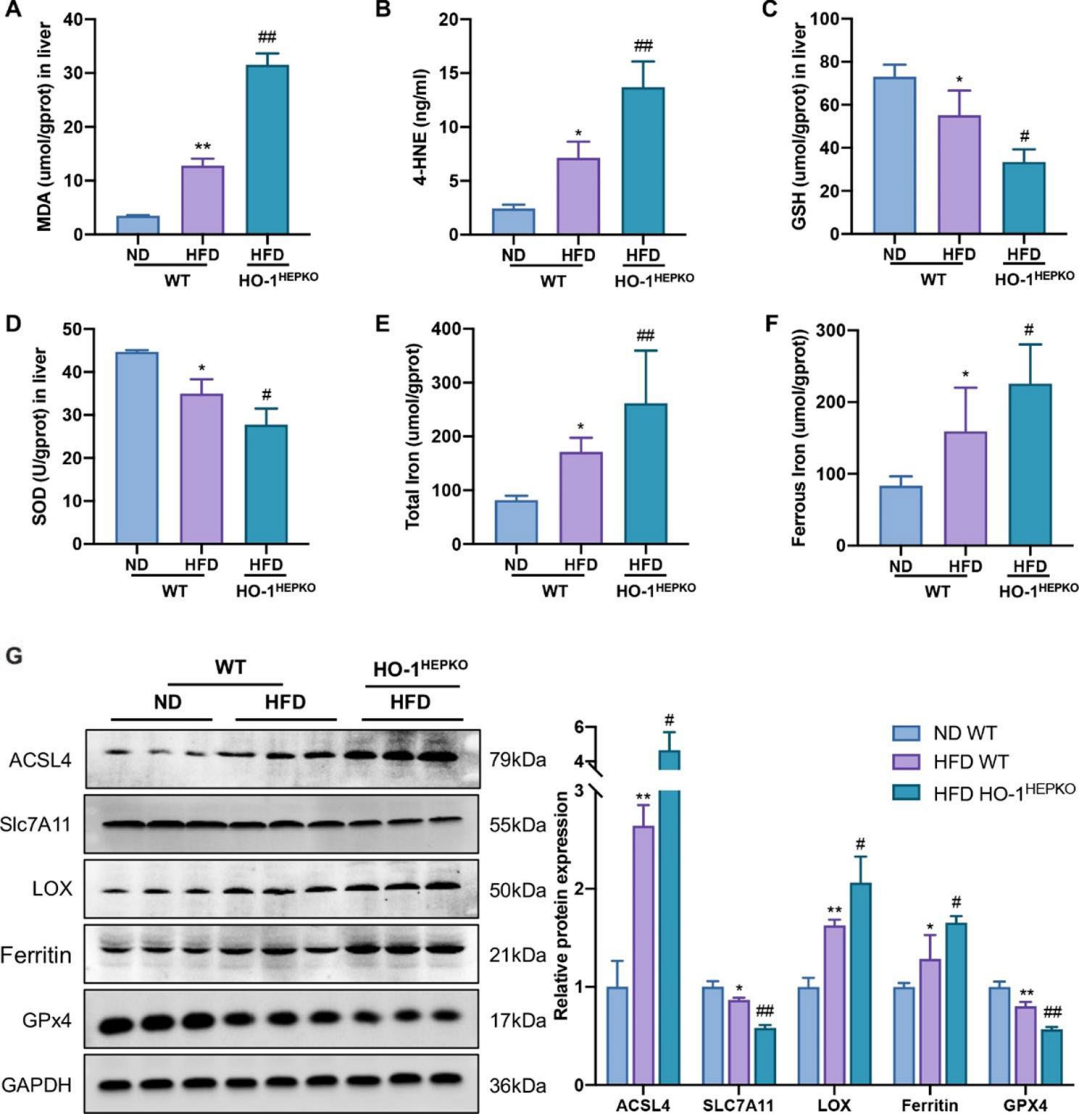
HO-1 deficiency exacerbates hepatic ferroptosis in mice with NASH. (**A, B, C, D**) The levels of MDA, 4-HNE, GSH, and SOD in the liver. (**E, F**) The levels of total iron and ferrous iron contents in the liver. (**G**) Western blot analysis of SLC7A11, GPX4, Ferritin, ACSL4, LOX in liver tissue. Values are the mean ± SD (n = 6 per group). *P < 0.05, **P < 0.01 compared to the WT ND group; *#P <* 0.05, *##P* < 0.01 compared to the WT HFD group

To explore whether HO-1 deficiency exacerbated the peroxidation of hepatic lipids induced by HFD in vivo, we detected MDA and 4-HNE content and GSH and SOD levels. As shown in Fig. 3A and D, the concentrations of MDA and 4-HNE were significantly upregulated, and the GSH and SOD levels were downregulated in the WT HFD group compared with those in the WT ND group. Furthermore, compared to the WT HFD group, the concentrations of MDA and 4-HNE in the HO-1^HEPKO^ HFD group were meaningfully higher. Additionally, the HO-1^HEPKO^ HFD group had significantly lower levels of GSH and SOD than the WT HFD group.

In mouse liver tissue, we assessed ferroptosis-related protein expression and iron content to reveal the role of HO-1 in hepatic ferroptosis. We found that HFD treatment increased the total and ferrous iron contents. However, the changes were more substantial in the HO-1^HEPKO^ HFD group (Fig. 3E F). In addition, HFD treatment significantly suppressed SLC7A11 and GPX4 protein levels compared to ND treatment. However, SLC7A11 and GPX4 levels decreased more after HO-1 knockout in the HO-1^HEPKO^ HFD group. Moreover, the increased expression of Ferritin, ACSL4 and LOX in the WT HFD group was exacerbated by hepatic conditional HO-1 knockout in the HO-1^HEPKO^ HFD group (Fig. 3G). Therefore, HO-1 deficiency increased hepatic ferroptosis severity in mice with NASH, suggesting a protective role of HO-1.

### HO-1 mediates lipid droplet accumulation and hepatic lipid peroxidation in vitro

**Fig. 4:**
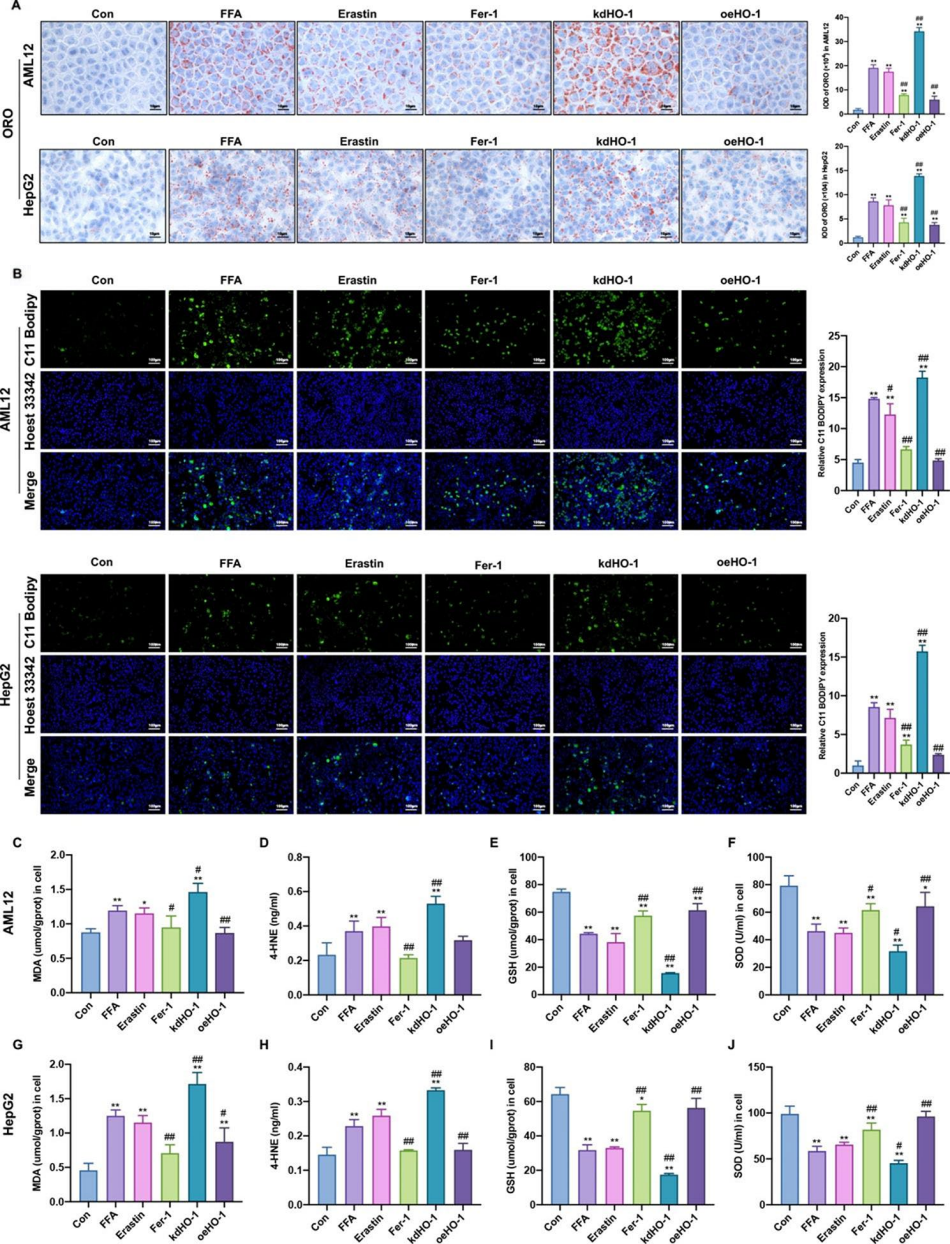
HO-1 reduced lipid droplet accumulation and hepatic lipid peroxidation in AML12 and HepG2 cells. (**A**) Representative images of Oil Red O analysis of lipids in AML12 and HepG2 cells (scale bar = 10 μm). (**B**) C11 BODIPY 581/591 staining in AML12 and HepG2 cells (green). Nuclei were stained using Hoechst 33,342 (blue) (scale bar = 100 μm). The levels of (**C, G**) MDA, (**D, H**) 4-HNE, (**E, I**) GSH, and (**F, J**) SOD in AML12 and HepG2 cells. Values are the mean ± SD. *P < 0.05, **P < 0.01 compared with the control group; #*P* < 0.05, ##*P* < 0.01 compared with the FFA group

To further explore the effect of HO-1 on ferroptosis, we cultured AML12 and HepG2 cells with FFA to simulate the accumulation of lipid droplets and hepatic lipid peroxidation. The confirmation of HO-1 knockdown and HO-1 overexpression in AML12 and HepG2 cells by both qPCR and Western blotting is shown in Supplementary Figure S2, and the siRNA sequences are shown in Table S1. As shown in Fig. 4A, the FFA and erastin groups accumulated significantly more lipids than the controls. Compared with the FFA group, the kdHO-1 group aggravated lipid accumulation, while the Fer-1 and oeHO-1 groups decreased lipid accumulation in AML12 and HepG2 cells. The immunofluorescence findings (Fig. 4B) revealed that lipid ROS accumulated in the FFA and erastin groups, which was in agreement with the Oil Red O staining results. Moreover, HO-1 knockdown aggravated lipid ROS levels in steatotic cells, while Fer-1 treatment and HO-1 overexpression reduced lipid ROS production (Fig. 4B). Compared with the control group, the MDA and 4-HNE contents were apparently increased, and the GSH and SOD levels were reduced in the FFA and erastin groups (Fig. 4 C-4 J). Furthermore, the increase in MDA and 4-HNE and the decrease in GSH and SOD in steatotic hepatocytes were augmented by HO-1 knockdown, while these effects were reduced following Fer-1 treatment and HO-1 overexpression (Fig. 4 C-4 J). Thus, the findings indicated that HO-1 protected against the peroxidation of hepatic lipids caused by FFA.

### HO-1 decreases ferrous iron content and inhibits hepatic ferroptosis in steatotic hepatocytes

**Fig. 5:**
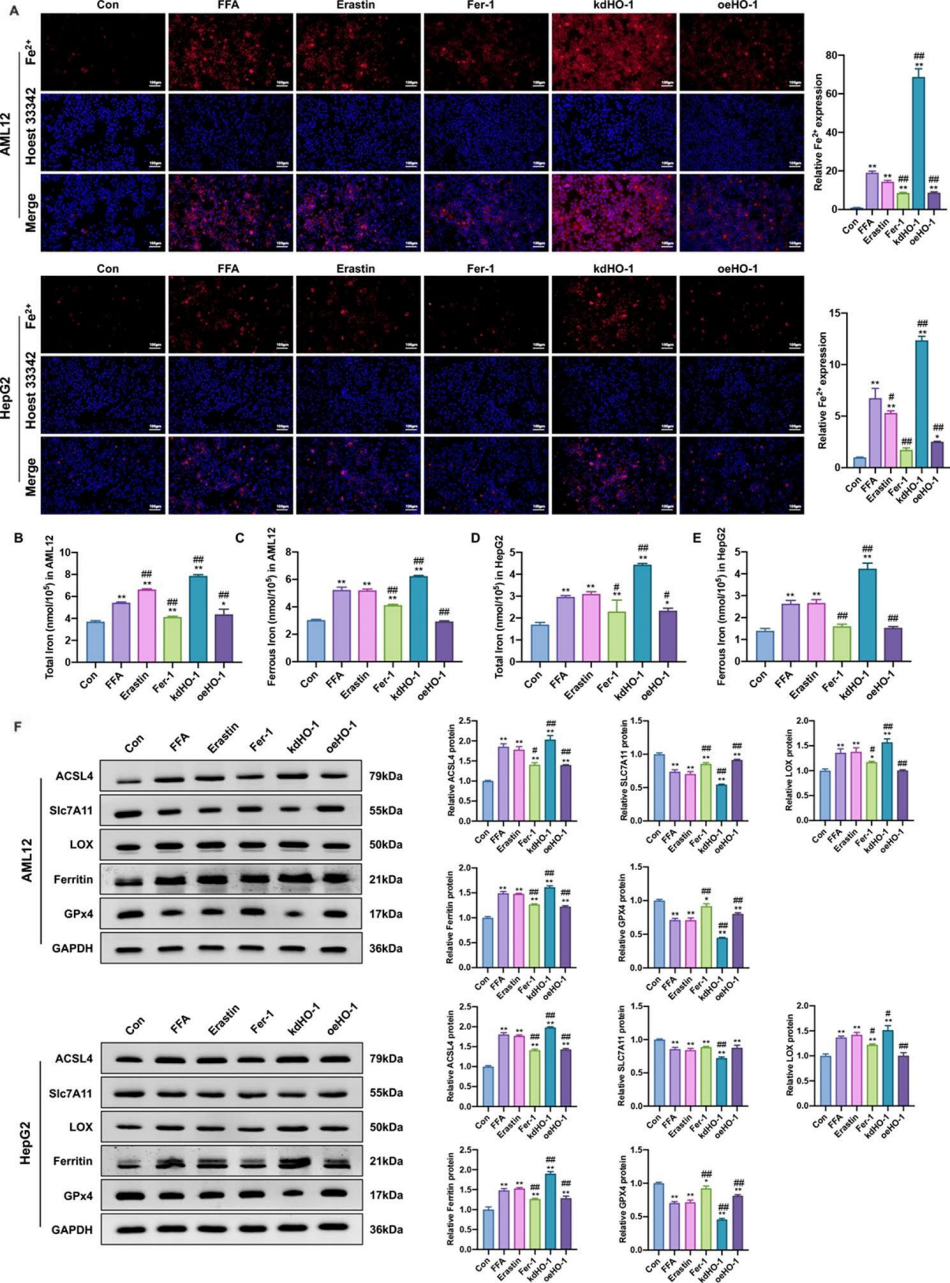
HO-1 inhibited hepatic ferroptosis in steatotic hepatocytes. (**A**) Ferrous iron staining (red) in AML12 cells. Nuclei were stained using Hoechst 33,342 (blue) (scale bar = 100 μm). (**B**, **C, D, E**) The total iron and ferrous iron levels in AML12 and HepG2 cells. (**F, G**) Western blot analysis of SLC7A11, GPX4, Ferritin, ACSL4, LOX in AML12 and HepG2 cells. Values are the mean ± SD. *P < 0.05, **P < 0.01 compared with the control group; #*P* < 0.05, ##*P* < 0.01 compared with the FFA group

To determine whether HO-1 inhibits ferroptosis in vitro, we examined iron levels and the expression of ferroptosis-related proteins. Compared to the control group, the FFA and erastin groups had a marked increase in iron content. Additionally, the high iron content was alleviated by Fer-1 supplementation and HO-1 overexpression but aggravated by HO-1 knockdown (Fig. 5A and E). SLC7A11 and GPX4 were significantly downregulated, while Ferritin, LOX and ACSL4 were expressively upregulated in the FFA and erastin groups compared to the control group. Moreover, Fer-1 supplementation and HO-1 overexpression markedly increased SLC7A11 and GPX4 expression and decreased ferritin, LOX, and ACSL4 expression in steatotic hepatocytes, while HO-1 knockdown produced the opposite results (Fig. 5F). Therefore, these data indicated that FFA treatment induced hepatic ferroptosis in vitro, which could be alleviated by HO-1 overexpression.

### HO-1 suppresses ferroptosis by inhibiting the NF-κB pathway in vivo and in vitro

**Fig. 6:**
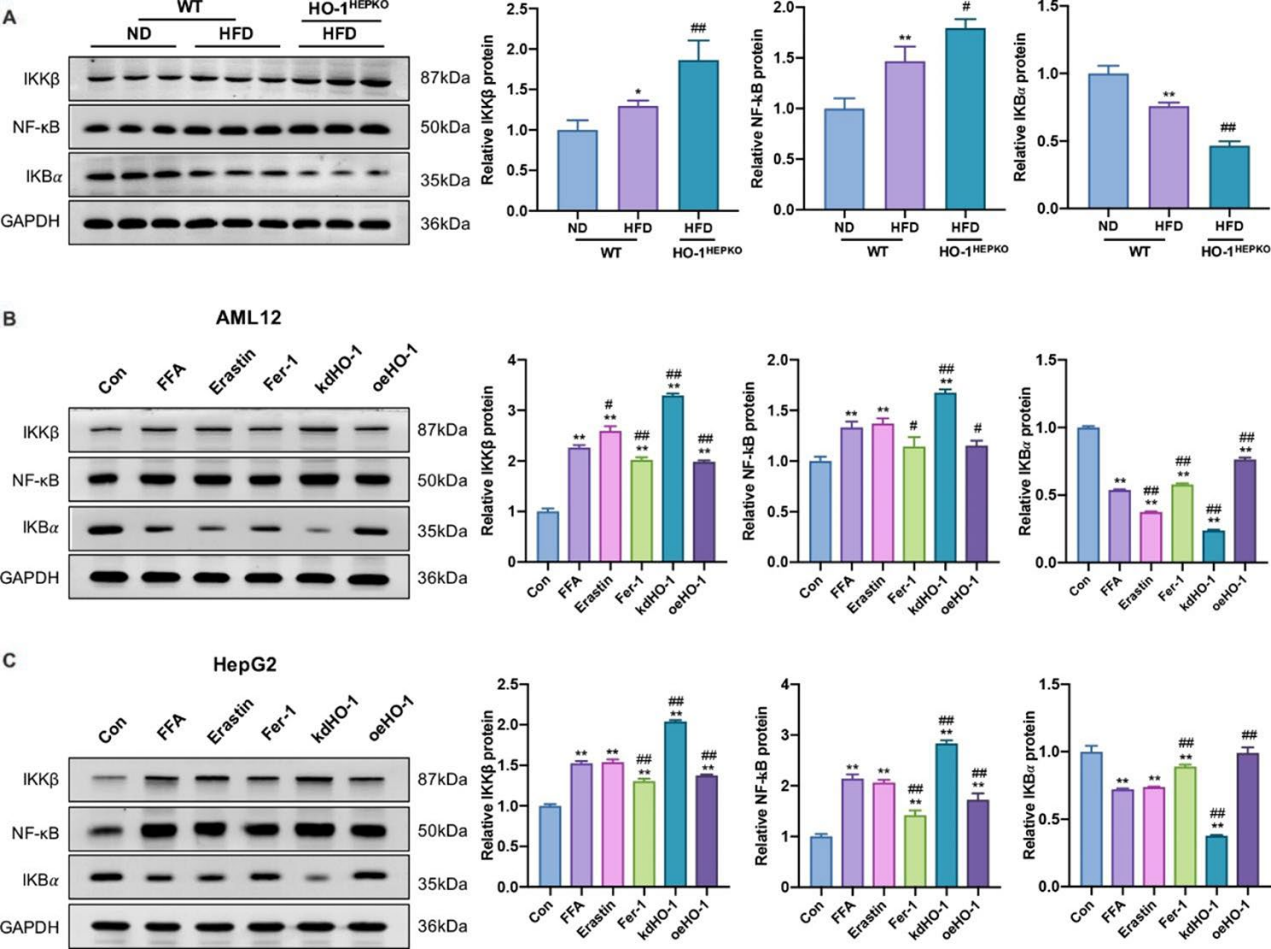
HO-1 mediates ferroptosis by inhibiting the NF-κB signaling pathway in vivo and in vitro. (**A**) Western blot analysis of NF-κB, IKBα, and IKKβ in liver tissue. Values are the mean ± SD (n = 6 per group). *P < 0.05, **P < 0.01 compared to the WT ND group; *#P <* 0.05, *##P* < 0.05 compared to the WT HFD group. (**B, C**) Western blot analysis of NF-κB, IKBα, and IKKβ in AML12 and HepG2 cells. Values are the mean ± SD. *P < 0.05, **P < 0.01 compared with the control group; #*P* < 0.05, ##*P* < 0.01 compared with the FFA group

We evaluated NF-κB signaling pathway protein expression to better understand how HO-1 regulates ferroptosis in NASH. As shown in Fig. 6A, the increased expression of NF-κB and IKKβ and the decreased expression of IKBα in HFD-fed mice were augmented by HO-1 deficiency. Furthermore, compared to the control group, NF-κB and IKKβ expression was significantly upregulated in the FFA and erastin groups, and HO-1 knockdown augmented the increased protein expression (Fig. 6B C). In contrast, Fer-1 supplementation and HO-1 overexpression markedly decreased protein expression in vitro. However, the expression of IKBα contrasted with that of NF-κB and IKKβ in vitro (Fig. 6B C). The data demonstrated that HO-1 mediated ferroptosis at least partially via the NF-κB pathway.

### The expression levels of HO-1, ferroptosis-related key factors, and the NF-κB pathway in NASH patients

**Fig. 7:**
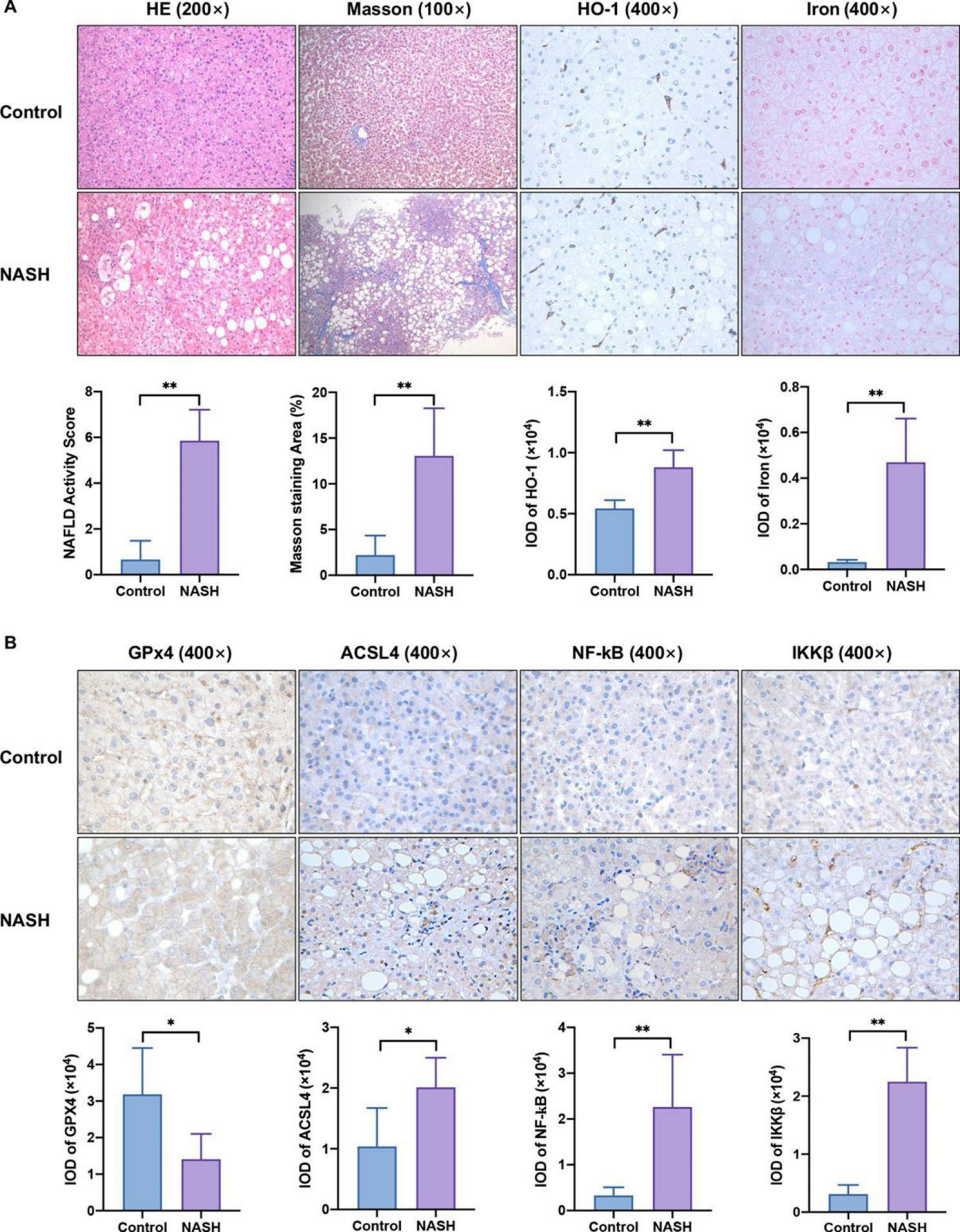
Validation of the expression of HO-1, ferroptosis-related key factors, and the NF-κB pathway in patient livers. (**A**) Representative H&E and Masson staining (×200 magnification) from healthy controls (n = 6) and NASH patients (n = 21). (**A, B**) Expression of HO-1, iron, GPX4, ACSL4, NF-κB, and IKKβ proteins was determined by immunohistochemistry staining (×400 magnification) in human liver tissues. The results are the mean ± SD. *P < 0.05, **P < 0.01 compared with the control group

To verify whether ferroptosis is involved in NASH, we first measured the expression of ferroptosis biomarkers in patients with biopsy-confirmed NASH. The demographic characteristics of the subjects are shown in Table 1. As shown in the H&E, Masson and Perls staining of liver sections from NASH patients (Fig. 7A), hepatocyte macrosteatosis and ballooning, necroinflammation and fibrosis, and ferric ion in the hepatic lobules were observed. Moreover, IHC results showed substantially increased expression of HO-1, ACSL4, NF-κB, and IKKβ in human liver biopsy samples from patients with NASH (Fig. 7A and B). Importantly, GPX4 expression was decreased in NASH patients compared to healthy controls (Fig. 7B). Thus, these data suggested that increased HO-1 may modulate NASH by alleviating patient ferroptosis via the NF-κB pathway.

**Table 1:** Demographical characteristics of subjects

Variables	Control	NASH	*χ2/t/Z*	*P*
**Gender (male/female)**	4/2	18/3	1.122	0.289
**Age (years)**	33.83 ± 10.94	34.62 ± 13.57	-0.130	0.898
**BMI (kg/m2)**	21.51 ± 1.47	27.06 ± 2.93	-4.445	0.000
**ALT (U/L)**	16.00 (9.60–23.00)	98.00 (57.00-152.00)	-3.413	0.001
**AST (U/L)**	20.35 ± 4.59	56.33 ± 26.57	-5.906	0.000
**TC (g/L)**	3.50 ± 0.86	4.80 ± 1.16	-2.534	0.018
**TG (g/L)**	1.08 ± 0.44	1.88 ± 1.26	-1.507	0.144
**Fasting GLU (mmol/L)**	4.75 (4.63–5.56)	5.32 (5.15–5.55)	-1.284	0.199

BMI, body mass index; ALT, alanine aminotransferase; AST, aspartate aminotransferase; TC, total cholesterol; TG, triglyceride; GLU, glucose; NASH, nonalcoholic steatohepatitis

## Discussion

Our study showed that HFD consumption or FFA treatment accelerated lipid deposition, ROS production, iron deposition, and lipid peroxidation, which triggered hepatic ferroptosis and led to hepatic steatohepatitis and fibrosis. We mechanistically revealed that HO-1 overexpression improved NASH by activating the system Xc-/GSH/GPX4 axis and inhibiting iron deposition-dependent oxidant stress-mediated ferroptosis. In addition, the findings demonstrated that HO-1 could mediate ferroptosis via the NF-κB signaling pathway.

The widely accepted theories interpreting the etiology of NAFLD are the “two-hit hypothesis” and the “multiple parallel hits hypothesis”, both of which consider that multiple factors simultaneously trigger the progression of NAFLD, including oxidative stress, lipid peroxidation, and inflammatory cytokine production [13]. Thus, inhibiting lipid peroxidation and oxidative stress is an effective NASH treatment strategy. Notably, it was previously identified that HO-1 could antagonize oxidative stress by catalyzing antioxidant production in hepatocytes [13]. However, suppression of HO-1 expression exacerbates the progression from hepatic steatosis to hepatic fibrosis [17]. Consistent with other findings, we found that elevated lipid accumulation, liver injury, and hepatic fibrosis were aggravated in the livers of HO-1^HEPKO^ HFD mice. Furthermore, the overexpression of HO-1 could reduce hepatic damage and lipid peroxidation in vivo and in vitro. Although HO-1 has a beneficial function in NASH, the underlying mechanisms require further investigation.

Ferroptosis, a new form of controlled cell death associated with lipid peroxidation in an iron-dependent manner, has been proven to play a cardinal role in NASH [12]. Previous studies have reported the ability of HO-1 to protect against iron homeostasis [14] and potential oxidative damage during stress [12, 13, 17]. However, the pathogenic link and mechanism by which HO-1 inhibits ferroptosis to improve NASH need elucidation. In the current research, we observed that HO-1 deficiency could exacerbate ROS accumulation, lipid peroxidation, and iron deposition caused by HFD-fed mice and FFA-treated cells. In contrast, in line with Fer-1 treatment, HO-1 overexpression alleviated these changes. It has also been previously established that one of the primary causes of ferroptosis is lipid peroxidation [5, 10, 11, 18]. When an external stimulus occurs, freakishly high contents of free radicals increase lipid peroxidation and oxidative stress and then promote the advancement of IR and the eventual progression of NASH [5]. MDA and 4-HNE are the two main adducts among the secondary lipid peroxidation products, which could circuitously reflect the extent of peroxidation injury and the different stages of NASH [5, 19]. SOD, a potent antioxidant enzyme, plays a crucial role in the oxidative stress response by catalyzing the formation of oxygen and hydrogen peroxide from superoxide anion radicals, which helps to keep the level of ROS in balance [19]. Notably, our results showed that HO-1 overexpression reduces MDA and 4-HNE. Additionally, it increased the content of SOD, suggesting that HO-1 could significantly inhibit ROS accumulation and proinflammatory factor production and improve the levels of antioxidative stress in NASH models. Thus, this could be one of the primary ways by which HO-1 reduces liver damage caused by ferroptosis.

Previous studies have indicated that HO-1 is a modulator of iron signaling, but the exact function of HO-1 in mediating iron metabolism in NASH is still unclear [16, 18]. Generally, iron homeostasis and metabolism are meticulously orchestrated by an elaborate regulatory system [20, 21], and the labile iron pool is regulated at a particularly low level. In contrast, extra iron can be exported through ferroportin or stored in ferritin. Superoxide, however, may cause the release of Fe^2+^ from the FeS cluster, heme, ferritin, and the iron pool in conditions of extreme oxidative stress [19]. Consequently, free Fe^2+^ (labile iron) is highly oxidative and is prone to give rise to damaging oxygen free radicals via the Fenton reaction, which can cause iron toxicity to nucleic acids, proteins, and membrane lipids to promote lipid peroxidation [18, 22]. We found that HO-1 knockout or knockdown in the study could significantly increase the total and ferrous iron content. In contrast, HO-1 overexpression led to a decrease in iron content *in vivo and in vitro*. Likewise, a few studies have indicated that the increased expression of HO-1 in the NASH model [17] may accelerate heme catabolism and lead to enhanced Fe^2+^ production [14]. However, studies have shown that ferroptosis-induced liver injury is initiated by the accumulation of free nonheme iron in liver tissue [23]. Notably, HO-1 can contribute to inhibiting ferroptosis by recycling free iron to a compensatory increase in heme synthesis. More importantly, HO-1 can also cause the consequent induction of ferritin synthesis, which plays a crucial role in protecting cells against oxidative damage [14]. It also appears that ferritin synthesis following lipid peroxidation may be above what is required to store excess iron, preventing iron from participating in Fenton reactions [14, 24, 25]. Thus, this would help to explain the cytoprotective effects of HO-1 induction, which is critical for iron homeostasis.

System Xc-, composed of SLC7A11 and SLC3A2, mainly contributes to specific cystine uptake and antioxidant GSH generation [11]. In addition to scavenging free radicals in the cell and maintaining balance, System Xc- also serves as the cofactor of GPX4, which participates in reduction reactions [26]. Our findings revealed lower SLC7A11, GSH, and GPX4 in the livers of HFD mice and FFA-treated hepatocytes, suggesting an imbalance in the System Xc-/GSH/GPX4 pathway in NASH models. Likewise, in accordance with previous studies [27–30], stimulating HO-1 overexpression could directly stimulate the System Xc-/GSH/GPX4 axis, which is widely acknowledged as a key target to prevent lipid peroxidation [31] and can utilize GSH to interfere with the lipid peroxide chain reaction by reducing the production of complex hydroperoxides [11]. The lipid peroxidation chain reaction can also occur during the liberation of polyunsaturated fatty acids, which are oxidized through a catalytic pathway involving ACSL4 and LOX. However, the lipid peroxides generated by the process that drives ferroptosis can be affected by GPX4 [10]. Herein, the activation of HO-1 to GPX4 or directly to ACSL4 and LOX could decrease lipid peroxidation and subsequently alleviate ferroptosis.

Several signaling pathways associated with lipid peroxidation and iron metabolism regulate the progression of NASH and ferroptosis [22]. Likewise, the NF-κB pathway plays a necessary role in ferroptosis. Studies have shown that the induction of iron overload and lipid peroxidation motivates the oxidative stress-responsive transcription factor NF-κB, which, in turn, increases the production of proinflammatory cytokines such as IL-6, IL-1β, and TNF-α, promoting inflammatory responses that further culminate in hepatocellular injury [5, 32–35]. Notably, the present study emphasized the capability of HO-1 to meaningfully decrease NF-κB and IKKβ expression, which further illustrates its protective properties against hepatic iron toxicity and the associated pathological conditions. In line with other investigations, studies have demonstrated that HO-1 can block the breakdown of IKBα, hence suppressing NF-κB nuclear translocation [36]. Probing into the latent processes underlying HO-1’s verified antioxidant and anti-inflammatory effects in NASH iron deposition found that the NF-κB pathway is implicated, which may help to understand the probable mechanism by which HO-1 modulates ferroptosis.

### Strengths and limitations

After a thorough literature review, it was found that this is the first report that clarified regulatory effect of hepatocyte HO-1 to NASH ferroptosis in both of antioxidant stress and regulation of iron metabolism, and possible regulatory pathways.

The precise and integrated signaling pathways and their binding sites between HO-1 and NASH ferroptosis need further investigation.

## Conclusion

Our findings confirmed that hepatic ferroptosis resulted in excess iron deposition and that lipid peroxidation in NASH could be diminished by HO-1 overexpression, at least partially via the NF-κB pathway. Hence, elucidating the regulatory effect of HO-1 on ferroptosis could provide a different perspective and a new therapeutic target for NASH.

## Electronic supplementary material

Below is the link to the electronic supplementary material.

Supplementary Material 1

Supplementary Material 2

Supplementary Material 3

## Data Availability

The datasets used during the current study are available from the corresponding author on reasonable request.

## Abbreviations

NAFLD: Nonalcoholic fatty liver disease
NASH: Nonalcoholic steatohepatitis
HO-1: Heme oxygenase 1
HO-1HEPKO: Hepatocyte conditional HO-1 knockout
HFD: High fat diet
ALT: Alanine aminotransferase
AST: Aspartate aminotransferase
TC: Total cholesterol
TG: Triglyceride
IL-6: Interleukin-6
IL-1β: Interleukin-1 beta
TNFα: Tumor necrosis factor-alpha
LOXL2: Lysyl oxidase homolog 2
α-SMA: Alpha-smooth muscle actin
TGFβ: Transforming growth factor beta
MDA: Malondialdehyde
4-HNE: 4-hydroxynonenal
GSH: Glutathione
SOD: Superoxide dismutase
SLC7A11: Heterodimeric protein containing a light chain
GPX4: Glutathione peroxidase 4
ACSL4: Acyl-CoA synthetase long-chain family member 4
LOX: Lipoxygenase
FFA: Free fatty acids
NF-κB: Nuclear factor kappa-B
IKBα: NF-kappa-B inhibitor alpha
IKKβ: Inhibitor of kappa B kinase beta
