# Supplementary figures and images for: Heme oxygenase 1 alleviates nonalcoholic steatohepatitis by suppressing hepatic ferroptosis
Source: Lipids Health Dis. 2023 Jul 8;22:99. doi: 10.1186/s12944-023-01855-7 (PMC10329355; doi:10.1186/s12944-023-01855-7)

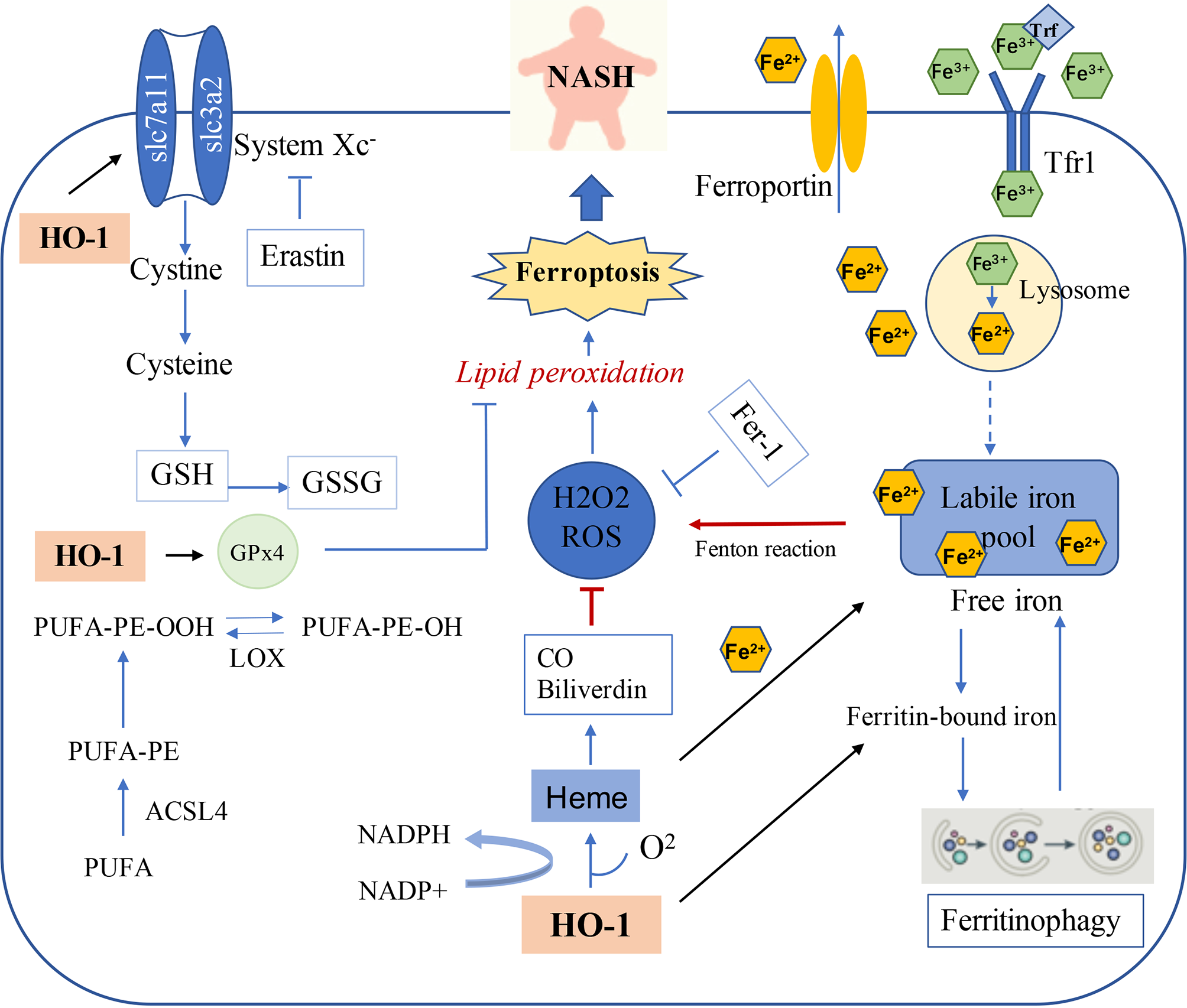

Supplement: Supplementary file 1 — Supplementary Material 1 [file 12944_2023_1855_MOESM1_ESM.tif]

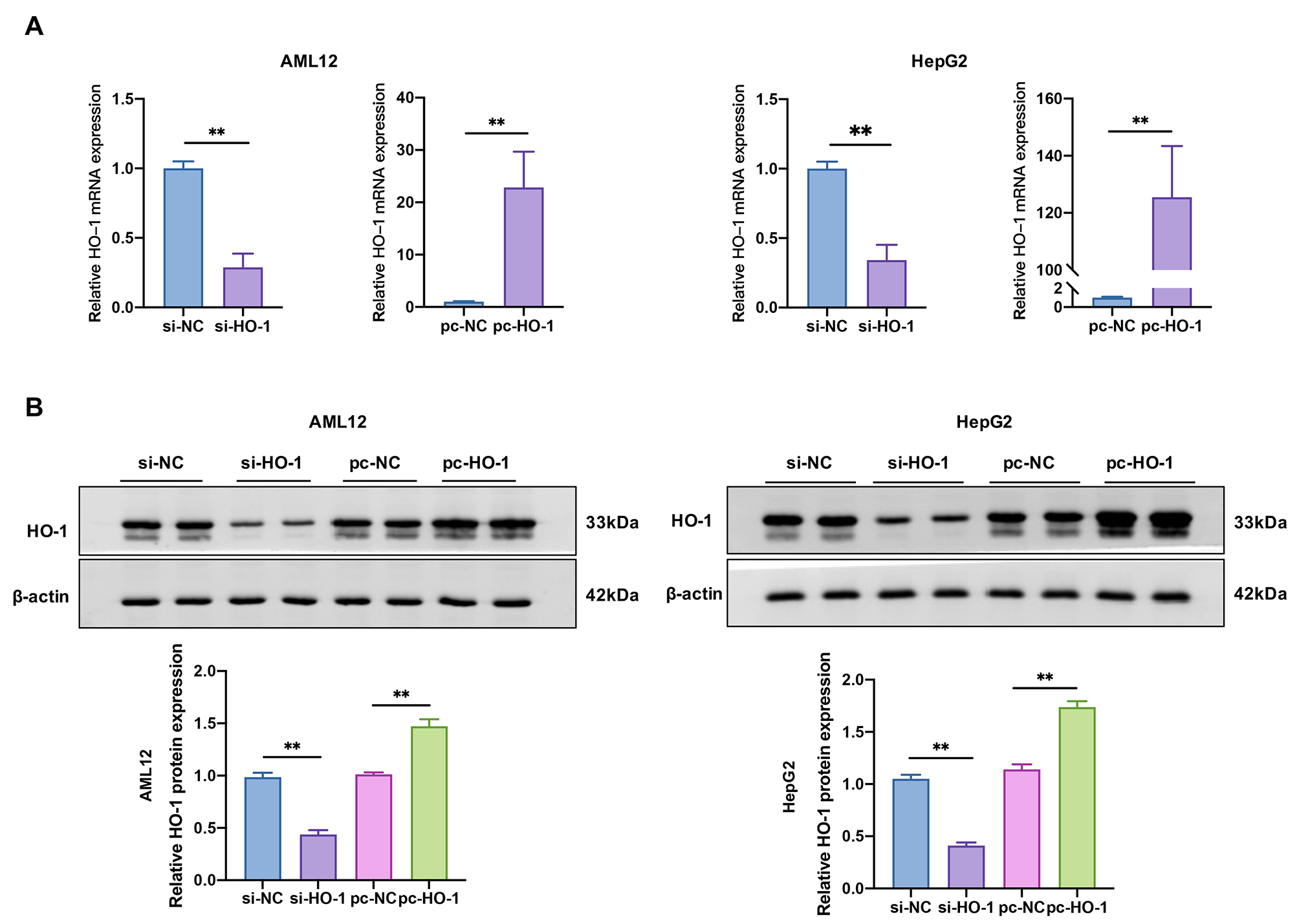

Supplement: Supplementary file 2 — Supplementary Material 2 [file 12944_2023_1855_MOESM2_ESM.tif]
