# Supplementary material for: Heme oxygenase 1 alleviates nonalcoholic steatohepatitis by suppressing hepatic ferroptosis
Source: Lipids Health Dis. 2023 Jul 8;22:99. doi: 10.1186/s12944-023-01855-7 (PMC10329355; doi:10.1186/s12944-023-01855-7)
Supplement: Supplementary file 3 — Supplementary Material 3 [file 12944_2023_1855_MOESM3_ESM.docx]

Table S1 the siRNA sequences and concentrations in AML12 and HepG2 cells

| Cells | Groups | Sequences |
| --- | --- | --- |
| AML12 | si-HO-1 | sense: 5′-CCGAGAAUGCUGAGUUCAUTT-3′  antisense: 5′-AUGAACUCAGCAUUCUCGGTT-3′ |
|  | si-NC  (Negative Control) | sense: 5′-UUCUCCGAACGUGUCACGUTT-3′  antisense: 5′-ACGUGACACGUUCGGAGAATT-3′ |
| HepG2 | si-HO-1 | sense: 5′-CAAUGGCCUAAACUUCAGATT-3′  antisense: 5′-UCUGAAGUUUAGGCCAUUGTT-3′ |
|  | si-NC | sense: 5′-UUCUCCGAACGUGUCACGUTT-3′  antisense: 5′-ACGUGACACGUUCGGAGAATT-3′ |
